# Increased risk of type I errors in cluster randomised trials with small or medium numbers of clusters: a review, reanalysis, and simulation study

**DOI:** 10.1186/s13063-016-1571-2

**Published:** 2016-09-06

**Authors:** Brennan C. Kahan, Gordon Forbes, Yunus Ali, Vipul Jairath, Stephen Bremner, Michael O. Harhay, Richard Hooper, Neil Wright, Sandra M. Eldridge, Clémence Leyrat

**Affiliations:** 1 Pragmatic Clinical Trials Unit, Queen Mary University of London, 58 Turner St, E1 2AB London, UK; 2 Department of Medicine, Western University and London Health Sciences Network, London, ON Canada; 3 Division of Epidemiology and Biostatistics, Western University, London, ON Canada; 4 Division of Primary Care and Public Health, Brighton and Sussex Medical School, Brighton, UK; 5 Division of Epidemiology, Department of Biostatistics and Medicine, University of Pennsylvania, Philadelphia, PA USA; 6 INSERM CIC 1415, CHRU de Tours, Tours, France; 7 London School of Hygiene and Tropical Medicine, London, UK

**Keywords:** Cluster randomised trials, Small-sample corrections, Degree-of-freedom corrections, Mixed-effects models, Generalised estimating equations

## Abstract

**Background:**

Cluster randomised trials (CRTs) are commonly analysed using mixed-effects models or generalised estimating equations (GEEs). However, these analyses do not always perform well with the small number of clusters typical of most CRTs. They can lead to increased risk of a type I error (finding a statistically significant treatment effect when it does not exist) if appropriate corrections are not used.

**Methods:**

We conducted a small simulation study to evaluate the impact of using small-sample corrections for mixed-effects models or GEEs in CRTs with a small number of clusters. We then reanalysed data from TRIGGER, a CRT with six clusters, to determine the effect of using an inappropriate analysis method in practice. Finally, we reviewed 100 CRTs previously identified by a search on PubMed in order to assess whether trials were using appropriate methods of analysis. Trials were classified as at risk of an increased type I error rate if they did not report using an analysis method which accounted for clustering, or if they had fewer than 40 clusters and performed an individual-level analysis without reporting the use of an appropriate small-sample correction.

**Results:**

Our simulation study found that using mixed-effects models or GEEs without an appropriate correction led to inflated type I error rates, even for as many as 70 clusters. Conversely, using small-sample corrections provided correct type I error rates across all scenarios. Reanalysis of the TRIGGER trial found that inappropriate methods of analysis gave much smaller *P* values (*P* ≤ 0.01) than appropriate methods (*P* = 0.04–0.15). In our review, of the 99 trials that reported the number of clusters, 64 (65 %) were at risk of an increased type I error rate; 14 trials did not report using an analysis method which accounted for clustering, and 50 trials with fewer than 40 clusters performed an individual-level analysis without reporting the use of an appropriate correction.

**Conclusions:**

CRTs with a small or medium number of clusters are at risk of an inflated type I error rate unless appropriate analysis methods are used. Investigators should consider using small-sample corrections with mixed-effects models or GEEs to ensure valid results.

## Background

Cluster randomised trials (CRTs) involve randomising groups of patients to different treatment arms. CRTs are often used when it is difficult to apply the intervention at the individual level, when there is a risk of contamination between treatment groups, or for logistical reasons. Patient outcomes from the same cluster tend to be correlated, meaning that they are more similar to the other patients in their cluster than they are to patients in other clusters. This violates the standard statistical assumption that all patients in a trial are independent of one another. The clustering must therefore be taken into account in the analysis. Failure to do so can lead to an increased risk of a type I error (finding a statistically significant treatment effect when no such effect exists) [1–7].

The most common methods of adjusting for clustering in the analysis of CRTs are: (1) a cluster-level analysis, in which a summary measure of the outcome is obtained for each cluster, and these summary measures are analysed using a linear regression model [1–3], (2) a mixed-effects model, in which individual patients are analysed using a regression model which includes a random-effect for cluster [1–3, 8], and (3) generalised estimating equations (GEEs), in which individual patients are analysed using a regression model which allows for correlation between patients in the same cluster [1–3, 9]. The cluster-level analysis approach can control the type I error rate even with a small number of clusters; however, it can lead to reduced power compared to mixed-effects models or GEEs; as such, these latter approaches are much more commonly used in practice [10].

However, both mixed-effects models and GEEs are based on asymptotic theory and, therefore, assume that there is a large number of clusters. When these methods are used without a large number of clusters they can lead to an increased type I error rate [1–3, 11–20]. For example, one simulation study examining the use of GEEs in CRTs with a small number of clusters found that the type I error rate was over 40 % with only four clusters, and even when the number of clusters was increased to 40, the type I error rate was still too high at 7 % [12]. It is difficult to know exactly how many clusters are required to implement these methods safely, as this can be influenced by factors such as the intraclass correlation coefficient, the outcome type, or variation in size between clusters. However, for CRTs, most sources recommend that at minimum 30–40 clusters are required for mixed-effects models and 40–50 clusters are required for GEEs [1, 2, 12, 19].

This requirement is problematic for CRTs, as most have fewer than 40 clusters [21–23]. Therefore, standard implementations of GEEs or mixed-effects models in these scenarios may lead to an increased type I error rate. However, a number of small-sample corrections have been proposed for these analytical methods to improve their performance with a small or medium number of clusters [11, 13–18, 20]. These small-sample corrections usually work by either increasing the estimated standard error of the treatment effect, or by changing the degrees-of-freedom used to calculate confidence intervals or *P* values for the treatment effect. They do not typically affect the estimated treatment effect directly (that is, the same treatment effect is estimated with or without the use of these small-sample corrections).

A number of these small-sample corrections have been evaluated in the context of CRTs [13, 16, 17]. Simulation studies have found that these corrections can improve the type I error rate, although not all corrections work well across all scenarios, and so the best correction to use may depend on the specific scenario. Furthermore, there has thus far been little evaluation with a very small number of clusters (e.g. fewer than 10), and there is little consensus on which methods work best in these scenarios. Although further research is required to determine the optimal methods of correction in various scenarios, it is clear that using a small-sample correction with a small number of clusters is an improvement upon standard, uncorrected methods [11, 13–20].

However, despite the benefits of these small-sample corrections, it is unclear whether those conducting CRTs with a small number of clusters routinely use analysis techniques which adequately account for the small number of clusters. A previous review of articles published between 1998 and 2002 in two specific journals found that many trials were not analysed using appropriate methods [10]. A more recent review of CRTs using GEEs found that only one out of 28 trials used a small-sample correction [12]. However, there is little current information available on the use of small-sample corrections for mixed-effects models, or what overall percentage of CRTs are using adequate analysis methods with a small number of clusters. The aims of this study were, therefore, to (1) conduct a simulation study to assess the benefits of using a small-sample correction in a CRT with a small number of clusters, (2) reanalyse a published CRT with only six clusters to compare the effects of small-sample corrections in practice, and (3) to conduct a review of published CRTs to estimate how many were at risk of an increased type I error rate due to an inappropriate method of analysis.

## Methods

### Simulation study

We conducted a simulation study to assess the benefits of using small-sample corrections in CRTs with a small number of clusters. The simulations were based on a two-arm, parallel group CRT with a continuous outcome and an equal number of patients in each cluster. We considered both small and large cluster sizes, where the small cluster size was based on a family (*n* = 5 per cluster) and the large cluster size was based on a hospital (*n* = 100 per cluster). For the small cluster size we used an intraclass correlation coefficient (ICC) of 0.15, and for the large cluster size we used an ICC of 0.01.

We generated continuous outcomes for each participant within a dataset using the formula:

$$ {Y}_{ijl}={\gamma}_{ij}+{\varepsilon}_{ijl}, $$

where *Y*_*ijl*_ is the outcome from the *l*^*th*^ (*l* = *1,…,m*) patient in cluster *j* (*j* = *1,…,k*) in arm *i (i* = *1,2)*, *γ*_*ij*_ is the cluster-specific random effect for cluster *j* in arm *i*, and *ε*_*ijl*_ is the individual residual error. *γ*_*ij*_ and *ε*_*ijl*_ were generated independently from the normal distributions *N(0, ρ)* and *N(0, 1* − *ρ)*, respectively. It follows that *Y*_*ijl* ~_*N(0, 1)*, with an ICC of *ρ*. Clusters were randomly divided into two equal-sized groups, one assigned to the intervention and the other to the control. This data generation method implies a treatment effect of 0 (i.e. that the treatment is not effective). We generated 50,000 datasets for each simulation scenario.

We evaluated two methods of analysis that were inappropriate with a small number of clusters, and three methods of analysis that were appropriate. Inappropriate methods of analysis were those that performed the analysis at the individual level without an appropriate small-sample correction. This included a mixed-effects linear regression model with no correction, and GEEs with no correction. Appropriate methods of analysis were those performed at the cluster level, and those performed at the individual level using an appropriate small-sample correction. This included a cluster-level analysis (with cluster-level summaries being based upon the mean outcome across all patients in a cluster), a mixed-effects linear regression model using a Satterthwaite degree-of-freedom correction, and GEEs using the small-sample correction proposed by Fay and Graubard [11].

For each scenario (5 patients per cluster and ICC = 0.15; 100 patients per cluster and ICC 0.01), we varied the number of clusters from 6, 10, 20, 30, 40, 50, 60, 70, 80, 90, and 100. We estimated the type I error rate for each setting by calculating the proportion of datasets with a statistically significant treatment effect at the 5 % level. Simulations were performed using R software version 3.2.0.

### Reanalysis of TRIGGER

We reanalysed data from the Trial in Gastrointestinal Transfusion (TRIGGER) [24–26] to assess the impact of an inappropriate method of analysis in practice. TRIGGER was a feasibility CRT that compared a liberal red blood cell transfusion strategy with a restrictive strategy for patients with acute upper gastrointestinal bleeding. TRIGGER recruited 936 patients from six clusters, with three clusters in each treatment group [25].

One of the main feasibility outcomes in TRIGGER was the number of red blood cell units transfused. We reanalysed the data using appropriate and inappropriate methods of analysis, and compared the results. Inappropriate methods of analysis were those that did not adjust for clustering, or performed the analysis at the individual level without an appropriate small-sample correction. The methods tested were a linear regression model that did not account for clustering, a mixed-effects linear regression model without a degree-of-freedom correction, and a linear regression model using GEEs that did not include a small-sample correction.

Appropriate methods of analysis were those performed at the cluster level, and those performed at the individual level using an appropriate small-sample correction. The methods tested were a mixed-effects linear regression model using a Satterthwaite degree-of-freedom correction, GEEs using a small-sample correction [11], and a cluster-level summary method of analysis. The cluster-level summary technique was originally used in TRIGGER. For this approach, the mean number of units of blood transfused in each cluster was calculated and included as an outcome in a linear regression model [26].

We included clusters in the model as a random intercept for mixed-effects models. We specified an exchangeable correlation structure within clusters for GEEs.

### Review of published CRTs

This review used a set of CRTs previously identified using a published electronic search strategy [27]. CRTs were eligible for inclusion if they were published in English in 2011. They were excluded if they were quasi-experimental; were pilot, feasibility, or preliminary studies; did not collect any data at the individual level; only assessed cost-effectiveness; or were not the primary report of the trial findings. Trials were identified from PubMed using a pre-specified search strategy [27]. We randomly selected 100 of the 132 eligible trials for inclusion in this current review using randomly generated numbers in the statistical software package Stata.

Data were extracted independently by pairs of reviewers. Any disagreements were resolved by a committee of three members through majority consensus (BK, GF, and CL). We extracted data related to the analysis of the primary outcome, including the number of clusters and participants included in the analysis, whether the clusters were accounted for in the analysis, and which method of analysis was used. We used the following strategy to identify the primary outcome: (1) if only one outcome was listed as being primary, we used this, (2) if either no outcomes or multiple outcomes were listed as being primary, we used the outcome that the sample size calculation was based on, and (3) if no sample size calculation was performed, we used the first outcome listed in the results section of the abstract.

We classified trials as at risk of an increased type I error rate if they used an ‘inappropriate’ method of analysis that did not control the error rate, or not being at risk if they used an ‘appropriate’ method of analysis that did control the error rate. Trials were classified as using an inappropriate method of analysis if the authors did not report adjusting for clustering in the analysis, or if they performed the analysis at the individual level (i.e. based on individual patient data) without reporting the use of an appropriate small-sample correction when there were fewer than 40 clusters. Small-sample corrections included any degree-of-freedom correction for mixed-effects models (such as Kenward-Roger or Satterthwaite) [15, 16] and any small-sample correction for GEEs [11, 14, 17, 18, 20].

Trials were classified as using an appropriate method of analysis if the authors performed the analysis at the cluster level (i.e. where patient data were aggregated at the level of the cluster), if they used an appropriate small-sample correction when performing the analysis at the individual level with fewer than 40 clusters, or if they performed the analysis at the individual level with 40 or more clusters.

We chose 40 clusters as the cut-off for a sufficient number of clusters to conduct an analysis at the individual level without using an appropriate correction as this is the number commonly recommended [1, 2, 10, 12, 19].

## Results

### Simulation study

**Fig. 1:**
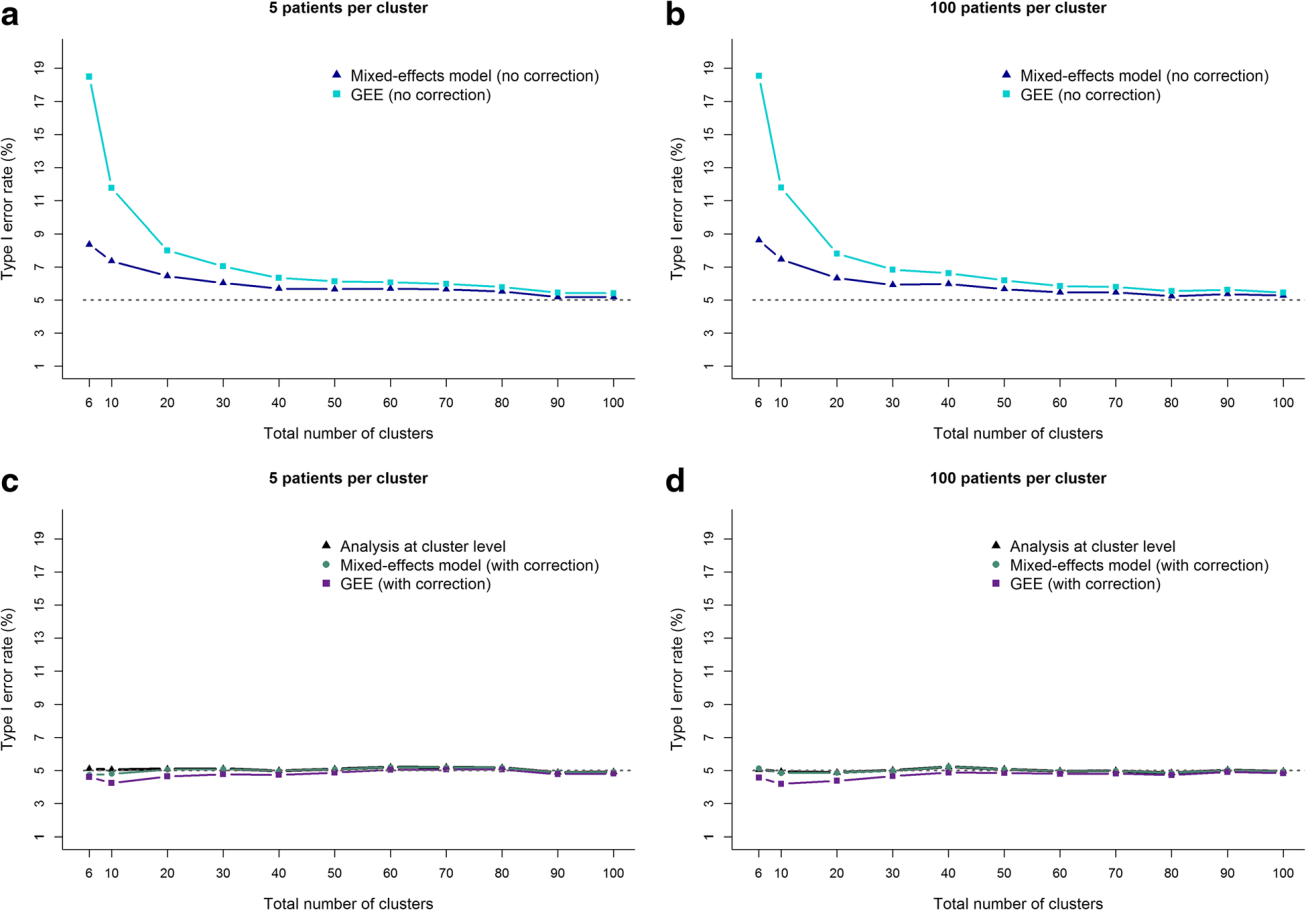
Association between type I error rate and number of clusters for ‘appropriate’ and ‘inappropriate’ methods of analysis. This figure presents the association between the total number of clusters and the risk of an inflated type I error rate for different methods of analysis. Panels **a** and **b** show the type I error rate for methods of analysis that are ‘inappropriate’ to use with a small number of clusters. Panels **c** and **d** show the type I error rate for methods of analysis that are ‘appropriate’ to use with a small number of clusters. The mixed-effects models in panels c and d used a Satterthwaite degree-of-freedom correction. The GEE models in panels c and d used the correction proposed by Fay and Graubard. Panels a and c represent a small cluster scenario (e.g. a family). Panels b and d represent a large cluster scenario (e.g. a hospital). The intraclass correlation coefficient (ICC; a measure of how correlated participants within a cluster are) was set as 0.15 for ‘small’ clusters (panels a and c), and as 0.01 for ‘large’ clusters (panels b and d). Data were estimated using simulation (50,000 replications per scenario)

Results are shown in Fig. 1. Using a mixed-effects model or GEEs without a small-sample correction led to inflated type I error rates when the number of clusters was small. With six clusters, mixed-effects models led to type I error rates of 8.4 % and 8.6 % for small and large cluster sizes, respectively, and GEEs led to an error rate of 18.5 % for both small and large clusters. Even with as many as 70 clusters, the type I error rates were still slightly too high; mixed-effects models led to error rates of 5.6 % and 5.5 % for small and large clusters, respectively, and GEEs led to error rates of 6.0 % and 5.8 %.

Conversely, using a cluster-level analysis, or a mixed-effects model or GEEs with a small-sample correction provided correct type I error rates across all scenarios, even with as few as six clusters (the type I error rate was below 5.2 % for all analyses across all simulation scenarios).

### Reanalysis of TRIGGER

The results of the reanalysis of TRIGGER are shown in Table 1. The estimated ICC was 0.01. All of the tested methods of analysis gave the same treatment effect. On average, 0.7 fewer red blood cell units were transfused in the restrictive group than in the liberal group.

**Table 1:** Reanalysis of the number of units of red blood transfused in the Trial in Gastrointestinal Transfusion (TRIGGER) trial

Analysis method	Is the analysis method appropriate for use with a small or medium number of clusters?	Difference in means^a^ (95 % CI)	*P* value
Unadjusted for clustering	No	−0.7 (−1.1 to −0.4)	<0.001
GEE (no correction)	No	−0.7 (−1.2 to −0.2)	0.01
Mixed-effects model (no correction)	No	−0.7 (−1.2 to −0.2)	0.01
GEE (with correction)	Yes	−0.7 (−1.8 to 0.4)	0.15
Mixed-effects model (with correction)	Yes	−0.7 (−1.4 to 0.0)	0.04
Cluster-level analysis	Yes	−0.7 (−1.6 to 0.3)	0.12

^a^The difference in means is presented as the restrictive transfusion policy minus the liberal transfusion policy

*CI* confidence interval, *GEE g*eneralised estimating equation

However, the results based on an inappropriate method of analysis (either ignoring clustering or performing an individual-level analysis without a small-sample correction) led to substantially smaller *P* values and narrower confidence intervals (CIs) than the results based on an appropriate method of analysis (performing a cluster-level analysis or using a small-sample correction in an individual-level analysis). The *P* values from the three inappropriate methods of analysis were all 0.01 or less. In contrast, the *P* values from the three appropriate methods of analysis were between 0.04 and 0.15.

Similarly, the 95 % CIs for the inappropriate techniques were all consistent with at least a small benefit for the restrictive group, as the upper bounds of the 95 % CIs across the three methods ranged between −0.4 and −0.2. In comparison, the upper bounds of the 95 % CIs from the three appropriate techniques were all consistent with little to no benefit, ranging from 0.0 to 0.4.

Using an inappropriate method of analysis in this trial with a small number of clusters thus overstated the evidence. Strong evidence of a reduction in the number of red blood cell units transfused in the restrictive arm was found, when in reality there was only borderline evidence of an effect.

### Review of published CRTs

Trial characteristics are shown in Table 2. The median number of clusters was 25 (IQR 15 to 44), 37 % of the trials had fewer than 20 clusters, and 69 % had fewer than 40 clusters. The primary outcome measure for most of the trials was either binary (53 %) or continuous (40 %).

**Table 2:** Characteristics of the included trials

	Trials (*n* = 100)
Number of clusters^a^ – median (IQR)	25 (15 to 44)
Number of clusters^a^ – number (%)	
4–9	14 (14)
10–19	23 (23)
20–29	18 (18)
30–39	13 (13)
40–49	11 (11)
50–79	7 (7)
80–99	4 (4)
100 or more	9 (9)
Patients per cluster^a^ – median (IQR)	31 (14 to 94)
Primary outcome – number (%)	
Continuous	40 (40)
Binary	53 (53)
Count	7 (7)
Accounted for clustering in analysis – number (%)	
Yes	86 (86)
No	10 (10)
Unclear	4 (4)

^a^Includes data from 99 trial reports, as one trial report did not state the number of clusters involved

*IQR* interquartile range

#### Methods of adjusting for clustering

The majority of the trials (86 %) accounted for clustering in the analysis, 10 % did not adjust for clustering, and 4 % did not report sufficient information to judge whether the analysis accounted for clustering or not (Table 2). Of the trials that did adjust for clustering, most (*n* = 77/86, 90 %) performed an individual-level analysis (Table 3) using either mixed-effects models (*n* = 46/77, 60 %) or GEEs (*n* = 21/77, 27 %). None of the trials that used mixed-effects models or GEEs reported using a small-sample correction. The number of clusters in the trial did not appear to influence the choice of analysis method.

**Table 3:** Number (%) of trials using different methods of analysis to account for clustering

	All trials (*n* = 86)	Trials with <40 clusters^a^ (*n* = 56)	Trials with 40+ clusters^a^ (*n* = 29)
Cluster-level or individual-level analysis (if adjusted for clusters)			
Cluster-level	8 (9)	6 (11)	2 (7)
Individual-level	77 (90)	50 (89)	26 (90)
Unclear	1 (1)	0 (0)	1 (3)
Analysis type if at individual level			
Mixed-effects model	46/77 (60)	31/50 (62)	15/26 (58)
GEE	21/77 (27)	14/50 (28)	6/26 (23)
Other	2/77 (3)	2/50 (4)	0/26 (0)
Unclear	8/77 (10)	3/50 (6)	5/26 (19)
Used a degree-of-freedom correction for mixed-effects model			
Yes	0/46 (0)	0/31 (0)	0/15 (0)
No	0/46 (0)	0/31 (0)	0/15 (0)
Unclear	46/46 (100)	31/31 (100)	15/15 (100)
Used a small-sample correction for GEEs – number (%)			
Yes	0/21 (0)	0/14 (0)	0/6 (0)
No	0/21 (0)	0/14 (0)	0/6 (0)
Unclear	21/21 (100)	14/14 (100)	6/6 (100)

^a^One trial was excluded as the number of clusters was not reported

*GEE* generalised estimating equation

#### Risk of inflated type I error rate

One trial did not report the number of clusters involved, so was excluded from the analysis. Table 4 shows that 65 % of the CRTs (*n* = 64/99) were at risk of an inflated type I error rate. These trials either used an individual-level analysis without reporting an appropriate correction despite having fewer than 40 clusters (50/64 cases, 78 %) or did not report using an analysis method which accounted for clustering (14/64 cases, 22 %). The remaining 35 trials were not classified as being at risk of an increased type I error rate because they used a cluster-level analysis (8/35 cases, 23 %) or because they used an individual-level analysis with 40 or more clusters (27/35 cases, 77 %).

**Table 4:** Number (%) of trials at risk of an inflated type I error rate

	Trials (*n* = 99^a^)
Assuming a minimum of 40 clusters is required for individual-level analysis with no correction (primary analysis)	
At risk of an inflated type I error rate – number (%)	
Yes	64 (65)
No	35 (35)
Reason for being at risk – number (%)	
Did not report adjustment for clustering in analysis	14/64 (22)
Individual-level analysis on <40 clusters without correction	50/64 (78)
Reason for not being at risk – number (%)	
Cluster-level analysis	8/35 (23)
Individual-level analysis with a large number of clusters (40+)	27/35 (77)

^a^One trial was excluded as the number of clusters was not reported

## Discussion

CRTs are commonly used in health care research and often include a small or medium number of clusters. This can make analysis of such trials challenging, as common methods of analysis such as mixed-effects models or GEEs can lead to inflated type I error rates in these situations. In our simulation study, we found that these methods, when used without an appropriate small-sample correction, led to inflated type I error rates, even for as many as 70 clusters. Conversely, using a cluster-level analysis, or a mixed-effects model or GEE with a small-sample correction led to valid type I error rates across all scenarios.

Our reanalysis of the TRIGGER trial found that the choice of analysis method had a large impact on conclusions about treatment efficacy. Mixed-effects models and GEEs without a correction led to very narrow confidence intervals and small *P* values, which overstated the strength of the evidence, indicating strong evidence of a treatment effect. Conversely, a cluster-level analysis, or a mixed-effects model or GEE with a small-sample correction led to much wider confidence intervals and larger *P* values, which more appropriately reflected the uncertainty around the size of the treatment effect estimate.

In our review of published CRTs, we found that most had fewer than 40 clusters (69 %). However, very few of these trials reported using an appropriate method of analysis. Overall, 65 % of the CRTs studied may have been at risk of an increased type I error rate. We note that the justification of 40 clusters being sufficient to use individual-level analyses without correction is more of an approximate guideline or rule-of-thumb rather than an absolute, and in some situations more clusters may be required; this is likely to depend on the specific trial characteristics (e.g. outcome type, ICC, variation in cluster size, etc.). In our simulation study we found that type I error rates were slightly inflated for as many as 70 or so clusters, indicating that perhaps small-sample corrections should be used for trials with even more than 40 clusters.

Our study had several limitations. In our simulation study, we considered only a small number of scenarios, and assessed only a small number of small-sample corrections. Therefore, on the basis of this study, we can recommend that trialists should adopt a small-sample correction when they have a small or medium number of clusters. However, we have not determined which methods of correction are the best for any particular situation. Other studies have explored a wider range of corrections, and should be used as the basis for choosing which specific correction to use [13, 16, 17]. However, we note that this area of research is ongoing, and in some cases the best correction to use may be unknown.

Secondly, although it is clear that using a small-sample correction performs better than not using a correction, there has been relatively little research into these corrections with a very small number of clusters (e.g. fewer than 10). There may, therefore, be some scenarios with a very small number of clusters in which small-sample corrections are not adequate to control the type I error rate. Including only a very small number of clusters also has other implications for the study’s validity [28]. Therefore, enrolling a larger number of clusters is preferable when feasible.

There were also some limitations to our review. It is possible that some investigators may actually have used a small-sample correction but did not report it, which would have affected our estimate of the proportion of trials using an inappropriate analysis method. This highlights the importance of clear reporting [29]. To allow readers to appropriately judge the validity of trial results, it is important for investigators to clearly state the method of analysis, including details on whether or not a small-sample correction was used. Secondly, we reviewed articles published in 2011; it is possible that small-sample corrections may have become more common since then.

## Conclusion

Small-sample corrections for mixed-effects models and GEEs are beneficial for CRTs; however, they are not often used in practice. Investigators should routinely use a small-sample correction when using mixed-effects models or GEEs with small or medium numbers of clusters, and should more clearly report whether or not these methods were used.

## Abbreviations

CRT: Cluster randomised trial
CI: Confidence interval
GEE: Generalised estimating equations
TRIGGER: Trial in Gastrointestinal Transfusion
